# Switch of Sensitivity Dynamics Revealed with DyGloSA Toolbox for Dynamical Global Sensitivity Analysis as an Early Warning for System's Critical Transition

**DOI:** 10.1371/journal.pone.0082973

**Published:** 2013-12-18

**Authors:** Tatiana Baumuratova, Simona Dobre, Thierry Bastogne, Thomas Sauter

**Affiliations:** 1 Systems Biology Group, Life Sciences Research Unit, University of Luxembourg, Luxembourg, Luxembourg; 2 Institute of Mathematical Problems of Biology, Russian Academy of Sciences, Pushchino, Moscow Region, Russia; 3 ISL, French-German Research Institute of Saint-Louis, Saint-Louis, France; 4 Université de Lorraine, CRAN, UMR 7039, Vandœuvre-lès-Nancy, France; 5 CNRS, CRAN, UMR 7039, Vandœuvre-lès-Nancy, France; 6 INRIA, BIGS, Vandœuvre-lès-Nancy, France; Georgia State University, United States of America

## Abstract

Systems with bifurcations may experience abrupt irreversible and often unwanted shifts in their performance, called critical transitions. For many systems like climate, economy, ecosystems it is highly desirable to identify indicators serving as early warnings of such regime shifts. Several statistical measures were recently proposed as early warnings of critical transitions including increased variance, autocorrelation and skewness of experimental or model-generated data. The lack of automatized tool for model-based prediction of critical transitions led to designing DyGloSA – a MATLAB toolbox for dynamical global parameter sensitivity analysis (GPSA) of ordinary differential equations models. We suggest that the switch in dynamics of parameter sensitivities revealed by our toolbox is an early warning that a system is approaching a critical transition. We illustrate the efficiency of our toolbox by analyzing several models with bifurcations and predicting the time periods when systems can still avoid going to a critical transition by manipulating certain parameter values, which is not detectable with the existing SA techniques. DyGloSA is based on the SBToolbox2 and contains functions, which compute dynamically the global sensitivity indices of the system by applying four main GPSA methods: eFAST, Sobol's ANOVA, PRCC and WALS. It includes parallelized versions of the functions enabling significant reduction of the computational time (up to 12 times). DyGloSA is freely available as a set of MATLAB scripts at http://bio.uni.lu/systems_biology/software/dyglosa. It requires installation of MATLAB (versions R2008b or later) and the Systems Biology Toolbox2 available at www.sbtoolbox2.org. DyGloSA can be run on Windows and Linux systems, -32 and -64 bits.

## Introduction

Recently the topic of system's critical transitions, or so-called tipping points, where a complex system performs a steep irreversible shift from one state to another, is getting an increasing attention [1]–[12]. Many systems in different domains of science are known to experience such abrupt shifts, like those in economics and global finance [13], [14], sociology [15], ecology and climate research [1], [6], [16], human physiology and medicine [17]–[19]. Recent theory suggests that critical transitions occur when a system passes its stability threshold [2], [9], [11]. While approaching the tipping point the system very slowly recovers from perturbations [2] and this effect is known in dynamical systems theory as ‘critical slowing down’ (CSD) [20], [21].

There were a number of studies targeting quantification of CSD and early prediction of the tipping points. Due to a limited capacities of modeling systems like climate or large ecosystems, model-based approaches for predicting the tipping points in such systems were considered dispensable [22] and the priority was given to data-driven approaches for quantifying the CSD by analyzing both model-generated and experimental time series data. Among possible measures of indication of system's irreversible change, there were proposed degenerate fingerprinting [21], [23], changes in spectral properties [24], increased autocorrelation [21], [22], [25] and variance [3], [19], [25]. These are attempts to find an alternative to a natural test for system's CSD which would be the study of how a system recovers from small experimental perturbations [22]. Although successful efforts were made recently to find experimentally early warnings of CSD using this approach [4], for some systems it might be irrelevant because of the difficulty to perform such experiments or when analyzing events happened in the past. In this context, model-based prediction of CSDs becomes more attractive. Examples of such predictions are shown in [10], [21], [22], [26]. However when favoring model-based methods of CSD prediction one should consider the non-linearity of the system structure and uncertainty of its parameter values which might seriously affect the resulting predictions.

In this paper we demonstrate the use of a widely known technique, parameter sensitivity analysis (PSA), to give early warnings of critical transitions of systems with bifurcations. PSA apportions the uncertainty in the model output to different sources of uncertainty in the model parameters which are characterized by large variation intervals. Local and global PSA methods are distinguished, where local ones study the effect of a single parameter variation (in the close vicinity to its nominal value) on the variation of a model output and global PSA shows how the overall variability of the model output can be associated with the variations of model parameters (considering the whole parameter state space (PSS)).

The MATLAB toolbox DyGloSA presented here allows the computation of dynamical global sensitivity indices (DGSIs) of ordinary differential equations (ODE) models and proposes that the switches in DGSIs behavior are early warnings of system's critical transitions. So far the usage of PSA methods for model-based predictions of CSD was very limited [21], in our opinion, because there were no automatized tool for performing dynamical global PSA. Various MATLAB toolboxes, like SBtoolbox2 [27] and SensSB [28], provide either the output of a single value of global sensitivity indices (GSIs) calculated for the given time interval [29]–[32], or dynamical, but local sensitivity indices (LSIs). However, neither dynamical LSIs nor snapshots of GSIs are capable to identify that a system approaches the critical transition: first ones provide a linear analysis of sensitivity dynamics and may be inappropriate for complex non-linear systems; the second ones provide only the state of sensitivity pattern at the end of the time interval and are not informative in terms of revealing the dynamics of sensitivity changes. Nevertheless, global dynamics is the underlying feature of all biological processes at all levels, and GSIs of biological models are also dynamically changing. Although some studies concerned the evaluation of GSIs over time [33]–[35], the lack of a convenient tool for computing dynamical GSIs reasoned developing of the DyGloSA toolbox. We suggest that the switch in dynamics of parameter sensitivities revealed by our toolbox provides the early warning that a system is approaching its critical transition. We demonstrate the efficiency of our toolbox by analyzing several models with multiple stability and predicting the time periods when systems can still avoid going to a critical transition by manipulating with certain parameter values.

## Results and Discussion

### Overview of the design and implementation of DyGloSA toolbox

DyGloSA is a MATLAB toolbox allowing the computation of dynamical GSIs of ODE models employing four GPSA methods [33]: two variance-based methods, namely Sobol's analysis of variance (ANOVA) [36], [37] and extended Fourrier amplitude sensitivity test (eFAST) [38]; partial rank correlation coefficients (PRCC) [39] and weighted average of local sensitivities (WALS) [40]. It is based on the respective functions for GPSA implemented in SB Toolbox2. For each of these GPSA methods DyGloSA includes a corresponding function for the computation of GSIs for parameters of the studied ODE model at each time step of the given time interval. Therefore the main input for DyGloSA functions is the ODE model given in SBML format. We briefly describe some additional inputs below, and the full description of the functions is included in the downloadable archive along with the toolbox and some examples. Outputs of the dynamical GPSA functions include the 3-D table of SIs of size (number of parameters)-by-(number of model states)-by-(time instances). In the downloadable archive we provide a sample program of how the SIs can be visualized and further in the Results section we explain how to analyze the obtained sensitivity profiles to examine the model behavior and find optimal times of adapting the system's performance in a desirable manner.

### Parallelized computation

As biological models are usually stiff and large, and accurate estimation of the numerical solutions require large number of sampling points of the PSS, the computation of SIs can be time consuming (from several hours to several days, depending on the size of the model and configuration of the PC used for simulations). In order to reduce of the computational time, we have equally implemented parallelized versions of the DGPSA functions (to run the parallelized functions the installation of the MATLAB Parallel Computing Toolbox is required). Thus, depending on the version of MATLAB installed and on the technical characteristics of the computer, the application of the parallelized functions can reduce the computation time from 4 to 12 times, or even more when using grid computing. The functions are also adapted to work on Windows 64-bit allowing the use of more than 4 GB of RAM. Each of the parallelized functions has the input parameter specifying the number of labs for computations to be parallelized on.

### Ranking of GSIs

We are interested in dynamical GPSA methods as they can determine the most sensitive parameters of the model. In the case of the selected representation of the DGPSA results, parameters are outputted in rows and model states in columns. So finding the most sensitive parameter is simply finding the row in the DGPSA result matrix having the highest value of sensitivity.

The raw values of sensitivity indices calculated with GPSA functions differ from one another depending on the selected PSA method. This makes direct comparison of the DGPSA results problematic. To overcome this we propose comparison of ranks of the SIs instead of the raw SIs values. DyGloSA includes a function which computes three types of ranks for the input 3-D matrix: a simple rank (SR), an average rank (AR) as proposed in [33], and an improved average rank (IAR) which we propose in this work. Ranks can be computed either row-wise or column-wise, depending on the input data matrix and the direction of rank calculation is given as an argument to the function.

The advantage of the average ranking over the simple ranking is the following: it allows avoiding different rank values for same or close raw values (Table 1, B. and C.). However, the similarly small and similarly high raw values cannot be distinguished from checking their ARs. To deal with this problem we propose an approach which we call improved average ranking, consisting of the following steps: (i) select the complete rows which have the same AR value, because elements of these rows might have either equally low values (as at row K4 of Table 1, C.) or equally high values (as at row K6 of Table 1, C.) of the raw sensitivity; (ii) find the global maximum of the raw sensitivity table (raw sensitivity value  = 100, Table 1, A.), locate its position (if several values are maxima, take the position of the first one) and select the column containing it (column M1); (iii) rank elements of this column and find the rank corresponding to the row with similar elements (rows K4 and K6 of our example); (iv) assign the above rank to all elements of the row. Modified so, the IARs of the rows with similar elements better represent their ranks in terms of the whole dataset, then ARs. The choice of the first global maximum at the step (ii) might slightly influence the final result; however the comparison of the elements of rows having similar AR values with any of global maxima will improve the result of average ranking because it will successfully distinguish low raw values from high raw values and therefore successfully update the average ranks.

**Table 1:** Different ranking of the sample table of sensitivity indices.

A.	Sensitivity values (raw)				B.	Simple ranks (SR)			
	M1	M2	M3	M4		M1	M2	M3	M4
K1	100	45	99	10	K1	4	2	3	1
K2	100	55	20	30	K2	4	3	1	2
K3	98	34	45	100	K3	3	1	2	4
K4	85	85	79	82	K4	3	4	1	2
K5	50	30	20	47	K5	4	2	1	3
K6	0	0	0	0	K6	1	2	3	4

C.	Average ranks (AR)				D.	Improved average ranks (IAR)			
	M1	M2	M3	M4		M1	M2	M3	M4
K1	3.5	2	3.5	1	K1	3.5	2	3.5	1
K2	3.5	3	1.5	2	K2	3.5	3	1.5	2
K3	3.5	1.5	1.5	3.5	K3	3.5	1.5	1.5	3.5
K4	2.5	2.5	2.5	2.5	K4	3	3	3	3
K5	3	2.5	1.5	3	K5	3	2.5	1.5	3
K6	2.5	2.5	2.5	2.5	K6	1	1	1	1

– parameters K4 and K6 seem to have similar sensitivity, however K4 is highly sensitive (according to raw values from A), and K6 is not sensitive at all; D. improved average ranks (IAR) of A – here the K4 is shown to be sensitive, and K6 is insensitive. Model states are located in columns (M1 to M4), parameters are located in rows (K1 to K6). A. Sample raw values of SIs; B. simple ranks (SR) of A - direct indication of sensitive parameters (rows) is not feasible; C. average ranks (AR) of A

### Warning of critical transitions

In order to detect time intervals when a system faces perturbations which might lead to a critical transition, we have designed the following algorithm which analyzes the sensitivity dynamics of the system:

- Perform the k-means clustering of the sensitivity indices per model state and time point (number of clusters  = 2). The composition of the top cluster at time *t* is recorded (parameter indices are sorted in decreasing order according to their values of sensitivity) and it shows to which parameter the given model state is sensitive at this time point. We apply one assumption which limits the number of the recorded elements of the top cluster: not more than N parameters might be sensitive at a time point *t*. This assumption is based on empirical observations that at steady states model states are usually sensitive to few parameters. If the top cluster is composed of more than N parameters, we record only N parameters with highest sensitivity values.
- Compare the current recorded composition of the top cluster with the one at a given time point, where the system was at a steady state, and count the number of differences observed.
- Select the time points where the difference in the recorded top cluster composition is equal to the maximal difference of the table (all the N top sensitive parameters must change), and consider these time points as those providing warnings of system's critical transition.
- We assume that more than M model states must have fully changed the composition of their top clusters at the given time *t* for the system to be considered to have a warning of its critical perturbation.

Both factors, N and M, are given as input parameters to the function of DyGloSA toolbox for detecting time intervals of critical perturbations. These parameters may be adapted to the investigated model, and their variation allows maintaining the balance between sensitivity of the system to the changing conditions and its functional robustness.

On the following figures demonstrating results of GPSA of models considered as case studies of DyGloSA application, grey regions represent the time intervals where the above mentioned algorithm indicated an early warning and the width of the grey region indicates how long the warning lasted. Within the marked early warning phase, a critical transition might occur. If a system was able to overcome the perturbation and return to its initial steady state, no critical transition occurred.

### Global PSA of systems with multiple stability

In order to illustrate the efficiency of DyGloSA in the early detection of system's critical transitions, several models were studied, including a model of the GATA1-PU.1 gene regulatory circuit [41], a three-species food chain model [42], [43], an apoptotic core model [44] and a model for immune cell interactions [45] which analysis is considered in details in the following sections. All the studied models are available in the downloadable package archive in the SBmodel format.

#### GATA1 – PU.1 gene regulatory circuit

The model describes a system containing mutual inhibition of two transcription factors, GATA1 and PU.1 and thus represents a bistable genetic toggle switch, previously published in [46]. The simple model without auto-regulation was considered here, where model states *X* and *Y* are defined by the relative activity levels of GATA1 and PU.1 respectively and the bistable behavior is given by the following system of ODEs: 
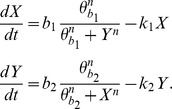



Parameters *b_1_* = *b_2_* = 1, *n* = 4 and *θ_b1_ = θ_b2_* = 0.5 are cross-inhibition parameters and *k_1_* = *k_2_* = 1 are decay constants, values are taken from [41]. The bistability of this system is well studied [41], [46], it was shown that the system has two stable steady states (one for *X*>>*Y* and another one for *Y*>>*X*) and one unstable saddle state situated on the separatrix between the two stable attractor states [41].

To demonstrate our approach for detection of system's critical transitions with DyGloSA, we have performed the following experiments: we have introduced at time  = 20 perturbations of initial conditions at different levels. Some perturbations are driving the system to a critical transition where PU.1 gene becomes active, whereas other perturbations are not severe enough and allow system's recovery to its beginning state where GATA1 is active.

**Figure 1:**
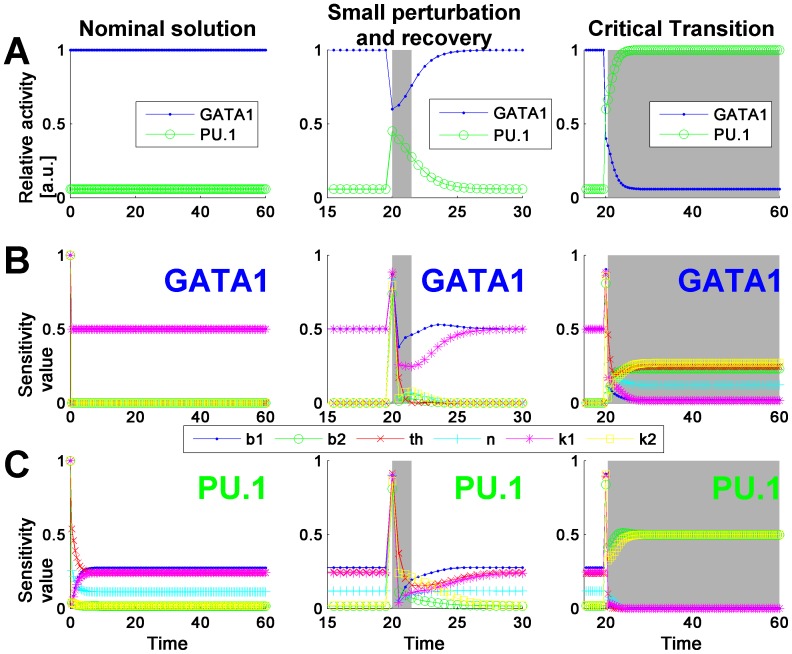
Dynamics of the model states and the sensitivity indices of the GATA1-PU.1 gene regulatory circuit model. The states dynamics is shown on line A; the sensitivity dynamics for GATA1 gene is shown on line B and for PU.1 gene – on line C. The left panel corresponds to the simulation providing the steady-state solutions. Middle panel corresponds to the introduced perturbation (concentrations of GATA1 and PU.1 were changed at time  = 20) and shows system's recovery after the perturbation. Plots of right panel show system's critical transition after significant perturbation of the initial conditions introduced at time  = 20. Time is given in an arbitrary unit. Grey regions indicate the periods when the system is perturbed and the sensitivity pattern differs from the one at the beginning of the simulations. Grey regions of middle panel suggest that the perturbation was non-critical and faded after 4 time instances when the system recovered to its previous state, while the grey region of the right panel indicates the warning of system's critical transition showing that system did not recover after the introduced perturbation.

The results shown on Figure 1 reveal that with the proposed approach of analyzing sensitivity dynamics calculated with DyGloSA, one can distinguish between critical (right panel of Figure 1) and non-critical (middle panel of Figure 1) perturbations. As discussed above, the grey regions on Figure 1 depict the warning period of criticality. In the case of relatively small perturbations the recovery of GATA1 activity is observed (middle panel of Figure 1) and when the perturbation is significant, the system experiences a critical transition which is indicated by the grey regions of Figure 1 (right panel).

**Figure 2:**
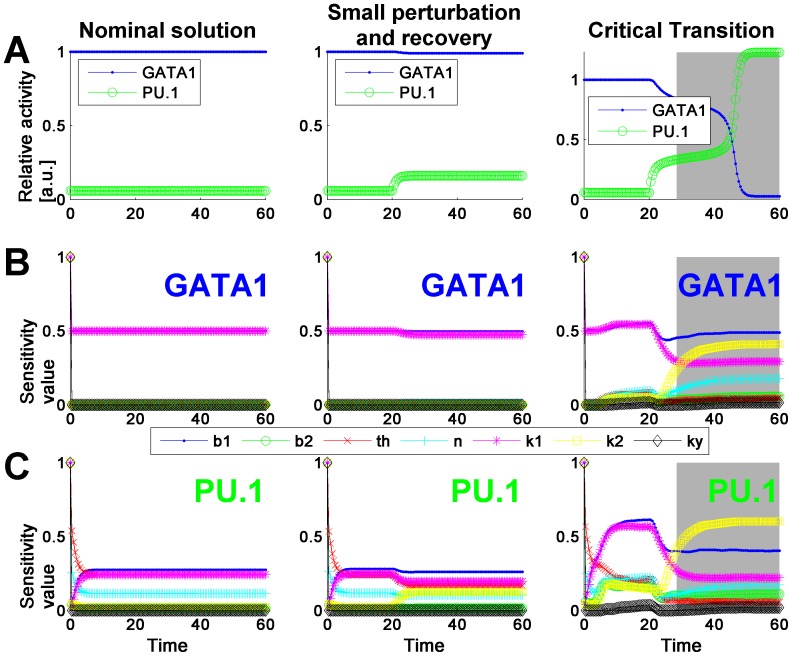
Dynamics of the model states and the sensitivity indices of the modified GATA1-PU.1 gene regulatory circuit model with added parameter *ky*. The states dynamics is shown on line A; the sensitivity dynamics for GATA1 gene is shown on line B and for PU.1 gene – on line C. The left panel corresponds to the simulation providing the steady-state solutions. Middle panel corresponds to the introduced perturbation (value of parameter *ky* was increased from 0 to 0.1 at time  = 20) and shows system's recovery after the perturbation. Plots of right panel show system's critical transition after significant perturbation of the parameter *ky* (value increased to 0.229) introduced at time  = 20. Time is given in an arbitrary unit. The grey region of the right panel provides the early warning of system's critical transition, because the observed transition (A, right panel) occurs at time ≈47 and the grey region begins at time ≈27.

Although the critical transition for this model was detected with the proposed approach, we did not observe an early warning as the critical perturbation was introduced with a step change of the state variables values. To check the ability of DyGloSA to provide early warnings of critical transitions, we have introduced a small modification in the model structure which lets the system slowly drift to a critical transition, by adding a parameter *ky* to the second equation: 
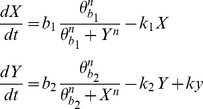



The addition of the parameter has no influence on the system's bistability pattern when its value is low (up to ∼0.15), and it causes the inactivation of the first steady state (X>>Y) when *ky* is higher than ∼0.2. The modified system was perturbed by increasing at time  = 20 the parameter *ky* to non-critical or to critical values (Figure 2, middle and right panels, respectively).

As follows from the plots of the middle panel of Figure 2, the introduced small perturbation did not have significant effect on the sensitivity pattern and so no warning region (grey region) is displayed. In contrast, in the case of relatively high perturbation (plots of the right panel of Figure 2) an early warning of the critical transition is given: critical transition, or actual switch of activity states, occurs at time ≈47, whereas the grey region, indicating the drastic change in the sensitivity pattern, starts at time ≈27.

#### Three-species food chain model

As another example of models which structures imply multiple stability when approaching certain values of parameters and state conditions we considered the food chain model from [42], originally published in [43]. The model describes a system including three interacting species: prey species X_1_, the only food source for predator species X_2_, which, in turn, are predated by second predator species X_3_. The model is given by a set of ordinary differential equations: 
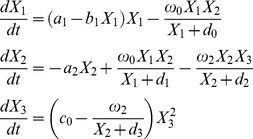
with parameter values taken from [43] and summarized in Table 2. Parameter *a_1_* is a bifurcation parameter and, as shown by authors of [43], depending on the value of *a_1_* the system may exhibit either two positive stable limit cycles or a stable limit cycle or one or two chaotic attractors. There are six equilibrium states of the system out of which only four have a significant effect on system dynamics [43].

**Table 2:** Parameters used in simulation of the three-species food chain model.

*a_1_*	*c_0_* = 0.038	*d_2_* = 10.0	*ω_1_* = 2.0
*a_2_* = 1.0	*d_0_* = 10.0	*d_3_* = 20.0	*ω_2_* = 0.405
*b_1_* = 0.06	*d_1_* = 10.0	*ω_0_* = 1.0	*ω_3_* = 1.0

**Figure 3:**
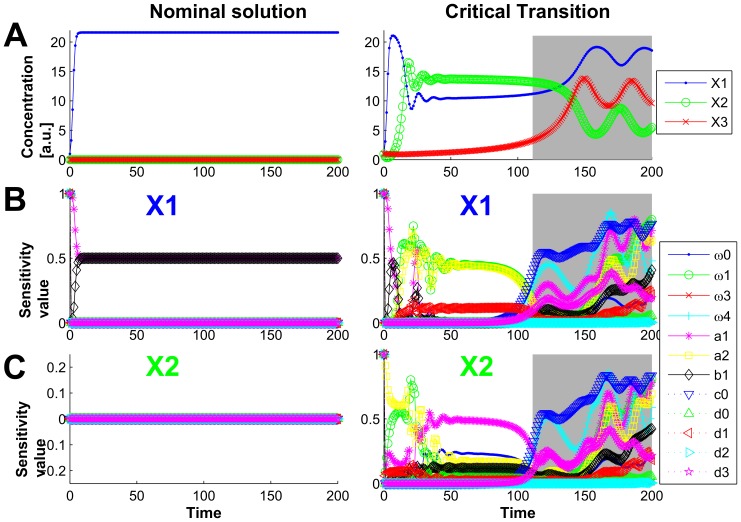
Dynamics of the model states and the sensitivity indices of the three-species food chain model. The states dynamics is shown on line A; the sensitivity dynamics for species X1 is shown on line B and for species X2 – on line C. The left panel corresponds to the simulation with values of initial conditions and the bifurcation parameter *a_1_* providing the steady-state solutions (*a_1_* = 1.297, *c_0_* = 0.029, IC = (1, 0, 0)). The right panel shows the dynamics of the state and sensitivity values for the bistable case (*a_1_* = 1.4, *c_0_* = 0.036, IC = (1, 1, 1)). Time is given in an arbitrary unit. Grey regions on plots of right panel provide the early warnings of system's critical transition: transition occurs at time ≈140, but the sensitivities for both model states change at time ≈110 (beginning of the grey region).

We considered two sets of values, including initial conditions (IC) and value of the bifurcation parameter, as described in [43] to analyze the system with DyGloSA: one set providing the equilibrium (*a_1_* = 1.297, IC = (1, 0, 0)) and the other one representing a bistable behavior (*a_1_* = 1.4, IC = (1, 1, 1)). The results of the analysis are shown of Figure 3. The steady state solutions (left panel of the Figure 3) of the model do not provide significant changes in the sensitivity dynamics. The increase of sensitivity with respect to the parameter *b_1_* and the decrease of sensitivity of the parameter *a_1_* within the first 10 time instances just follow the dynamics of the model solutions.

However, for the case of solutions with critical transitions (right panel of the Figure 3), the significant changes of the sensitivity dynamics can be observed. The sensitivity profiles indicate that the system is mostly sensitive to the changes in parameters *a_2_* and *ω_1_* (for both species *X_1_* and *X_2_*) within first 35 time units and for the *X_1_* state these parameters' sensitivities stay dominated, whereas for *X_2_* the parameter *a_1_* becomes more important (bifurcation parameter). The time region where the drastic changes in the sensitivity patterns for both states *X_1_* and *X_2_* begin is depicted by the grey region on the left panel of Figure 3: the parameters which were sensitive until this time interval become insensitive and new parameters become critical in terms of sensitivity (parameters *c_0_* and *ω_3_*).

We suggest that these significant changes in the parameter sensitivity dynamics indicate that the system approaches its critical transition. Indeed, the system has its tipping point approximately at time  = 140, however the dynamics of parameter sensitivities change earlier (around time  = 110) providing the early warning of the system's critical transition.

#### Apoptotic core model

**Figure 4:**
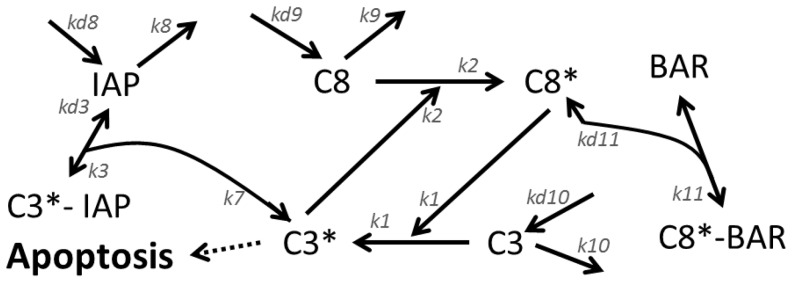
Summary of the key elements of the extended apoptotic core model describing the apoptotic pathway downstream of the death receptor.

**Figure 5:**
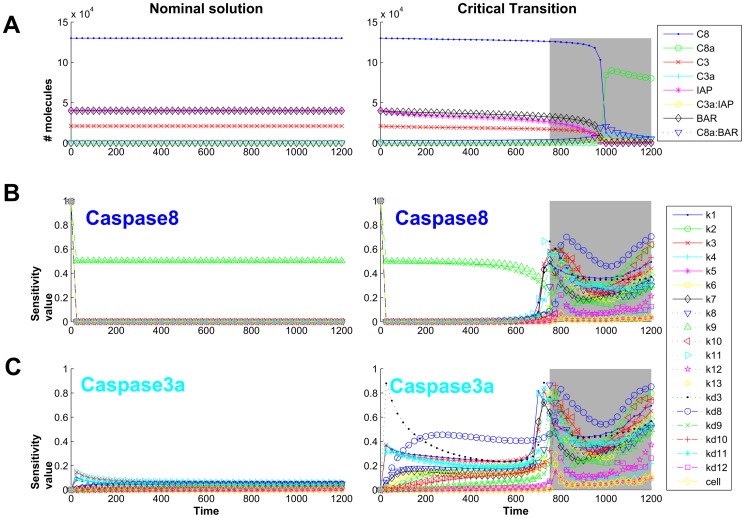
Dynamics of the model states and the sensitivity indices of the apoptotic core model. The states dynamics is shown on line A; the sensitivity dynamics for Caspase8 is shown on line B and for active Caspase3 – on line C. Left panel corresponds to the simulation providing the steady-state solutions (initial concentration of Caspase8 active  = 10 molecules). Right panel shows the dynamics of the state and sensitivity values for the case of switching between live and death states of cells at time  = 1000 (initial concentration of Caspase8 active  = 1000 molecules). Grey regions indicate the detected with DyGloSA early warning for the critical transition (the region begins at time ≈750).

We have also analyzed with DyGloSA the extended apoptotic core model from [44] (Figure 4). It describes the activation cascade of a family of aspartate-directed cysteine proteases, caspases, via stimulation of a death receptor. The full activation of caspases is an important step of the apoptosis because it leads to the destruction of cell membranes by cleaving their important regulatory and structural proteins [47]. It was shown [44], that the system becomes fully activated and two stable steady states (life and death) exist when the input signal exceeds a threshold of 75 molecules per cell, and the activation time depends on the input (initial concentration of C8*). Below this threshold only one stable steady state exists (life state) and no regime switch occurs (left panel of Figure 5, A). Consequently, for these initial conditions no change in sensitivity dynamics is observed (left panel of Figure 5, B and C).

We have also considered the case of C8*(0) = 1000 which leads to the full activation time of 1000 minutes (right panel of Figure 5, A). Analysis with DyGloSA revealed a drastic change of sensitivity dynamics of the model parameters at a time interval (700, 800) minutes (grey regions on the right panel of Figure 5, B and C).

#### Early- and late-sensitive parameters detection of the apoptotic core model

Our analysis shows that the parameters which were sensitive before this time point, k9 and kd9 are becoming non-sensitive and other parameters become sensitive for the time interval of approximately [700, 950] minutes.

**Figure 6:**
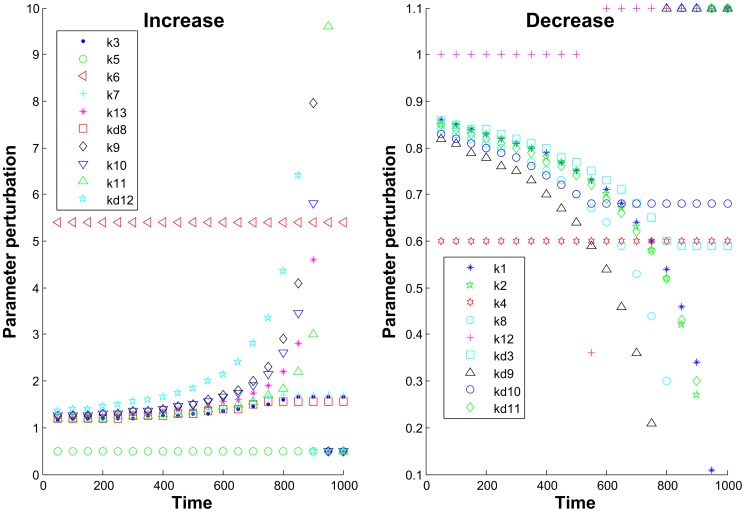
Time dependence of parameter variations necessary to prevent apoptotic state of the system. Left figure represents analysis of the parameters which values should be increased to prevent apoptosis and right figure shows parameters with necessary decrease in value. A value of variation equal to 0.5 for the left figure and to 1.1 for the right figure indicates that none variation of the certain parameter can prevent apoptosis, i.e. that the parameter has no effect on the system's behavior. Large perturbation values indicate that the system is losing its sensitivity to a certain parameter.

We suggest that the observed switch in parameter sensitivity dynamics can be considered as an early warning of the system's critical transition. Indeed, additional model simulations show that the 20% variation of the early-sensitive parameters (k9 and kd9) during the first 600 minutes does prevent the system to go to apoptosis (Figure 6). However, after the critical time point of 600 minutes, to enable system's recovery and return to the stable life state, the variation for these parameters must be about 100% of the nominal value. Similarly, for late-sensitive parameters, the relatively small variations (15–40%) of their nominal values enable system's recovery to a stable life state within appropriate times (between 700 min and 900 min) and on later times the much higher variations are needed to prevent apoptosis (400%–600% depending on the parameter) and for some parameters none variation of their values can prevent apoptotic state at all.

#### Immune cell interactions model

In the last case study presented here we analyzed the model in which interactions between immune cell population and the interaction-dependent properties of the immune system in homeostasis were investigated [45]. The interdependence of two cytokine populations, *X* and *Y*, mutually influenced via activatory and inhibitory cytokine productions, is described by the following system of ODEs: 
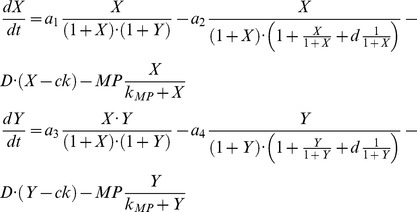



The model exhibits multi stable behavior with the model dynamics and steady states highly depending on the parameter values summarized in Table 3 [45]. There may be either a stable steady state solution representing the homeostatic concentrations of cytokines *X* and *Y* (H1), or multiple levels of homeostasis with two stable steady states representing stable low and high cytokines concentrations (H1 and H3) and one unstable state (H2), or the homeostatic stability is lost and limit cycles may appear leading to the possible oscillations in cytokines concentrations [45].

**Table 3:** Parameter values used in the immune cell interactions model.

*a_1_* = 0.251 (SH)	*a_2_* = 0.34 (SH)	*a_3_* = 0.12 (SH)	*a_4_* = 0.03 (SH)	*ck* = 0.25 (SH)
*a_1_* = 0.236 (TS)	*a_2_* = 0.3 (TS)	*a_3_* = 0.07 (TS)	*a_4_* = 0.03 (TS)	*ck* = 0.05 (TS)
*a_1_* = 0.2295 (Osc)	*a_2_* = 0.29 (Osc)	*a_3_* = 0.12 (Osc)	*a_4_* = 0.057 (Osc)	*ck* = 0.05 (Osc)
*d* = 0.5	*D* = 0.005	*MP* = 0.024	*k_MP_* = 0.6	

*a_1_*, *a_2_*, *a_3_*, *a_4_* and *ck* the model behavior is: SH - Stable Homeostasis, TS – Trigger Switch and Osc – Oscillations. Depending on the parameter values for

**Figure 7:**
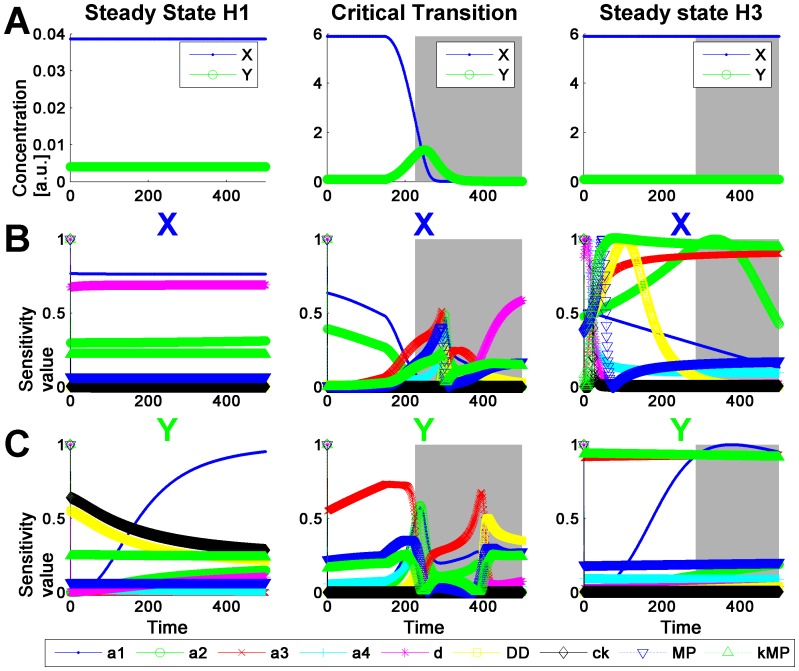
Dynamics of the model states and the sensitivity indices of the immune cell interactions model. The states dynamics is shown on line A; the sensitivity dynamics for gene X is shown on line B and for gene Y – on line C. The left panel corresponds to the simulation providing the steady-state solutions H1 (stable homeostasis). In middle panel the simulation with introduced perturbation leading to the critical transition are plotted (simulations start with ICs and parameter values corresponding to the steady state H3 (trigger switch) and at time  = 180 the parameter values are modified to those corresponding to the steady state H1 (stable homeostasis)). As displayed with the grey regions of the panel, sensitivity pattern provides the early warning of the critical transition. Right panel depicts the behaviors of model states (A) and sensitivity indices (B and C) in the case when the model is simulated with ICs and parameter values corresponding to the steady state H3 (trigger switch), but the range of parameter variations for DyGloSA functions was decreased by 10%. In this case although the model states do not exhibit any critical transition, the sensitivity pattern provides the early warning for it (presence of the grey regions on the plots of the right panel) because the perturbation range of the parameters covers the possible transition between the Trigger Switch state of the system and its Oscillatory behavior.

The analysis of the model with DyGloSA provided interesting results shown on Figure 7. The proposed method was able to fully capture the dynamics of the system: when simulated with the parameter values corresponding to the stable homeostasis (H1), the sensitivity pattern of the model shows very little dynamics and no warning of critical transitions is obtained (left panel of Figure 7).

A computational experiment was performed: starting the simulations with the initial conditions and parameter values corresponding to the steady state of stably high cytokines concentrations (H3), at time = 180 the parameter values were changed to those corresponding to H1. This provoked system's critical transition at time ≈290 with state *Y* becoming larger than state *X*, but the method of sensitivity pattern analysis provides the early warning at time ≈250 (grey region of middle panel, Figure 7). The beginning of the grey region is marking the time point when both model states fully changed the compositions of the top sensitive clusters. One might argue that this condition is too strict for a small system consisting of two model states only and that a warning should already be given when the sensitivity pattern of one state is changing. In this case for the middle panel example this would result in an early warning already around time ≈200 as the sensitivity pattern of state *X* is changing then. The respective input parameter of the DyGloSA function would have to be adapted accordingly.

Most interestingly, when the model is analyzed with DyGloSA with (i) 10% increase in parameter perturbation range, (ii) initial conditions and parameter values corresponding to H3 and (iii) no perturbation introduced (right panel of Figure 7), the sensitivity dynamics is still providing a warning for system's critical transition (grey regions on right panel of Figure 7), although no transition is observed for the model states (right panel of Figure 7, A). This result indicates that the bifurcation point is very close to the current parameter values and that even their small variations (covered by the 10% variation range) may lead to a critical transition. Indeed, the parameter values at Table 3 show very low difference between those specific for Trigger Switch mode and for Oscillations mode.

#### Autocorrelation and variance for immune cell interactions model

**Figure 8:**
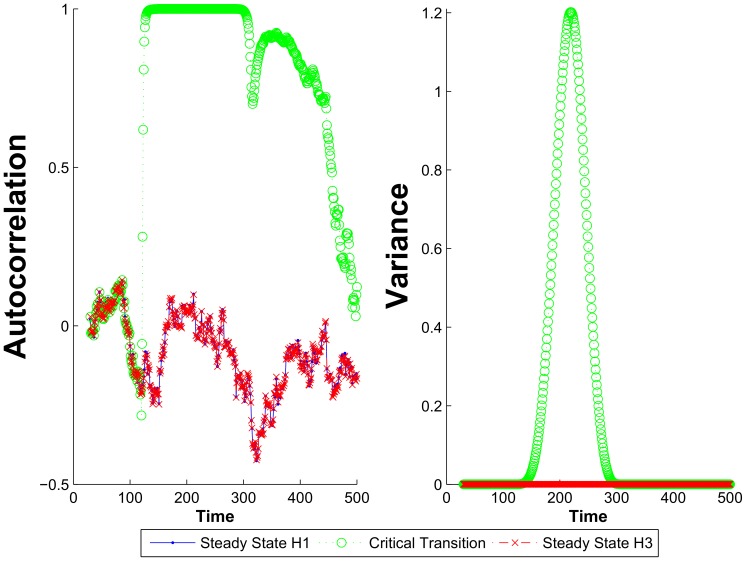
Autocorrelation and variance calculated for the immune cell interactions model. Both autocorrelation and variance increase for the case when model states exhibit critical transition and show no increase for the steady state solutions.

To bring the results of DyGloSA in predicting system's critical transitions to the context of the previously published works in the field, we have applied to our simulations data the two approaches which are widely used in literature for data-driven detection of critical transitions, namely analysis of autocorrelation and variance of the model-generated data[10], [22], [23], [25]. To calculate autocorrelation at lag 1 we have simulated the immune cell interaction model and added a randomly generated white Gaussian noise to the resulting values to overcome the problem of equality to zero of a denominator in case of flat curves. Variance and autocorrelation were calculated for the same resulting data using a sliding window approach (as proposed in [48]) and the results for the model are summarized on Figure 8.

As widely discussed in literature, when approaching the tipping point, a system slower recovers from small perturbation, and thus variance and autocorrelation increase. Our results also show the increase of autocorrelation and variance for the case with critical transition and no change for modeled steady states. Although we observe the increase of both autocorrelation and variance also a bit in advance than the critical transition occurs, this is not due to the method applied, but to the width of the sliding window. When the window is relatively narrow, no early warning can be given using the two methods.

The similarity of the two tests with DyGloSA results is partially explained by the methods behind two out of four GPSA functions: Fourrier Amplitude Sensitivity Test and Sobol's Analysis of Variances are variance-based methods, and the possibility of the global and dynamical analysis of wider parameter state space significantly extends the opportunities provided by classical analysis of autocorrelation and variance.

### Early warnings of critical transitions vs. system's fragility points

There should be distinguishing between cases when (i) a majority of the model states exhibit significant changes in GSIs dynamics, where the top early-sensitive parameters become insensitive and the leading sensitivity is taken over by previously insensitive parameters; and (ii) most model states show constant GSIs and a low number of model states have small changes in the GSIs dynamics including switches of sensitive parameters with low amplitude of the sensitivity response. The first case indicates system's critical transitions and is depicted as grey regions of the figures in the case studies above. The second case is revealing the fragile points of systems in terms of both parameters which variation can lead to the stability interruption as well as the model states which might consequently trigger the system's critical transition in response to the above parameter variation. Both fragility points and critical transitions are highly important for studying the system's dynamics and for providing clues for manipulation with the system's parameters to bring it to the desirable behavior.

### Conclusions

The main advantage of the proposed model-based approach for the prediction of early warnings for system's critical transitions is its simplicity and easiness in use. Once a model of a multi-stable process is achieved, it can be analyzed with DyGloSA and the dynamics of sensitivity pattern can be examined to check if there is a danger for the system to undergo critical transitions. Early warnings cannot be obtained from simple simulations of a model, because the simulations estimate the model solutions for a given set of nominal parameter values, either taken from literature, or obtained experimentally. Based on that, the critical transition can be revealed with simulations only when the nominal parameter set has already passed the no-return threshold and the system can't be pushed back to its desirable state. In contrast, using dynamical GPSA, we can predict whether or not a system is in danger of critical transitions because GPSA methods involve a certain variation of nominal parameter values. In DyGloSA this variation is flexible and user-defined, and can be specific to each of the parameters to ensure that all the relevant biological knowledge is respected during the analysis.

## Availability and Future Directions

DyGloSA is freely available for download at http://bio.uni.lu/systems_biology/software/dyglosa as a .zip archive which includes the toolbox functions together with the complete description, examples and installation guidelines. DyGloSA requires the installation of MATLAB (versions R2008b or later) and the Systems Biology Toolbox2 and SBPD available at www.sbtoolbox2.org. To use the parallelized functions, MATLAB Parallel Computing Toolbox has to be installed. DyGloSA can be run on Windows and Linux systems, both -32 and -64 bits.

Since the computation of sensitivity indices is seriously affected by the PSS sampling method, this fact can define the directions of DyGloSA development. We plan to implement other sampling methods, like quasi Monte Carlo sampling, which could improve the estimation of sensitivity indices for ANOVA and WALS methods.
